# Clinical Outcomes of Adult Patients Hospitalized with COVID-19 after Vaccination

**DOI:** 10.3390/tropicalmed6040175

**Published:** 2021-09-26

**Authors:** Markos Kalligeros, Fadi Shehadeh, Evangelia K. Mylona, Matthew Kaczynski, Saisanjana Kalagara, Eleftheria Atalla, Maria Tsikala Vafea, Eleftherios Mylonakis

**Affiliations:** 1 Infectious Diseases Division, Warren Alpert Medical School of Brown University, Providence, RI 02903, USA; markos_kalligeros@brown.edu (M.K.); fadi_shehadeh@brown.edu (F.S.); evangelia_mylona@brown.edu (E.K.M.); MKaczynski@lifespan.org (M.K.); saisanjana_kalagara@brown.edu (S.K.); elefthe100@gmail.com (E.A.); airam_t_v@hotmail.com (M.T.V.); 2 Department of Medicine, Warren Alpert Medical School of Brown University, Providence, RI 02903, USA

**Keywords:** COVID-19, SARS-COV-2, vaccination, breakthrough infections

## Abstract

Vaccination remains the most effective way to prevent COVID-19. The aim of the present study was to assess the incidence of COVID-19 hospitalizations after vaccination, as well as the effect of prior vaccination on hospitalization outcomes among patients with COVID-19. We analyzed and compared all consecutive patients, with or without prior vaccination, who were admitted to our hospital network due to COVID-19 from January to April 2021. Our primary outcome was to identify and describe cases of COVID-19 hospitalized after vaccination. We also utilized a multivariate logistic regression model to investigate the association of previous vaccination with hospitalization outcomes. We identified 915 consecutive patients hospitalized due to COVID-19 with 91/915 (10%) previously vaccinated with at least one dose of a COVID-19 vaccine. Utilizing our multivariate logistic regression model, we found that prior vaccination, regardless of the number of doses or days since vaccination, was associated with decreased mortality (aOR 0.44, 95% CI: 0.20–0.98) when compared to unvaccinated individuals. Our study showed that COVID-19 related hospitalization after vaccination may occur to a small percentage of patients, mainly those who are partially vaccinated. However, our findings underline that prior vaccination, even when partial, is associated with a decreased risk of death. Ongoing vaccination efforts should remain an absolute priority.

## 1. Introduction

Since the introduction of COVID vaccination, both clinical trial and real-world data have shown the high efficacy of COVID-19 vaccines in preventing SARS-CoV-2 infection and severe disease [1,2,3]. There is increasing interest in the study of breakthrough cases and limited data on patients who developed symptomatic COVID-19 requiring hospitalization after vaccination. Based on the latest MMWR report, from January to April 2021, around 10,000 cases of SARS-CoV-2 vaccine breakthrough infections were reported in the U.S., with ~10% of those cases requiring hospitalization [4]. In addition, as of 1 May, CDC has stopped monitoring vaccine breakthrough infections, and the attention has now transitioned only to hospitalized or fatal vaccine breakthrough cases [4]. To better understand vaccine breakthrough cases and their outcomes compared to unvaccinated COVID-19 cases, we analyzed and compared all consecutive patients, with or without prior vaccination, who were admitted to our hospital network due to COVID-19 from January to April 2021.

## 2. Materials and Methods

We analyzed all consecutive adult hospitalized patients (≥18 years old) with a primary diagnosis of COVID-19 and a positive RT-PCR who were admitted to Rhode Island Hospital (RIH), The Miriam Hospital, or Newport Hospital, in Rhode Island, between 1 January and 4 April 2021. Patients who were still hospitalized at the time of data extraction were excluded. This retrospective study was approved by the Institutional Review Board of RIH.

Two investigators (FS, MK) extracted the following deidentified variables of interest: age, gender, race, past medical history, medications received during hospitalization, mode of oxygen delivery, hospitalization outcome, and COVID-19 vaccination status (through our electronic immunization registry).

The primary endpoint was to identify breakthrough cases of COVID-19 and to assess the impact of previous vaccination on hospitalization outcomes. As a secondary endpoint, we compared hospitalization outcomes among previously vaccinated patients with COVID-19 to unvaccinated patients who were admitted with COVID-19 during the same period. For our analysis, we represented continuous measurements as medians (IQRs), and we compared them with the Mann–Whitney-Wilcoxon test. For categorical data, we used Pearson’s Chi-square test. We also utilized a multivariate logistic regression model (adjusting for demographics, comorbidities, dexamethasone, and Remdesivir use) to investigate the association of previous vaccination with hospitalization outcomes, namely ICU admission, need for invasive mechanical ventilation, and death/discharge to hospice. All analyses were performed using Stata v15. (Stata Corporation, College Station, TX, USA) and 95% confidence intervals and *p*-values are shown.

## 3. Results

### 3.1. COVID-19 Hospitalizations

We identified 915 consecutive patients hospitalized with a primary diagnosis of COVID-19 from 1 January to 4 April 2021. All patients had oxygen requirements upon admission. Among them, 91/915 (10%) had received at least one dose of a COVID-19 vaccine. Among vaccinated patients, 75/91 (82%) were hospitalized after receiving the first dose of a mRNA-based vaccine, and the majority (50/75, 67%) tested positive within the first 2 weeks after vaccination.

Interestingly, 16 patients were hospitalized after receiving either both doses of the BNT162b2 Pfizer-BioNTech or the Moderna mRNA-1273 COVID-19 mRNA-based vaccine or a single dose of the Ad26.COV2.S Janssen Biotech Inc. adenoviral vector vaccine. The majority of these individuals (14/16, 88%) tested positive within the first 2 weeks after vaccination (Table 1).

### 3.2. Outcomes of COVID-19 in Patients with Prior Vaccination

Most patients with prior vaccination (82/91, 91%) had a complete recovery, although 13/91 (14%) of vaccinated patients required ICU admission. Of note, 4/75 (5%) partially vaccinated and 4/16 (25%) patients who had received 2 doses of an mRNA-based vaccine or a single dose of the adenoviral vector vaccine died or were discharged to hospice (Table 2). All 4 patients who died after 2 doses of an mRNA-based vaccine or a single dose of the Ad26.COV2. S adenoviral vector vaccine tested positive within the first 2 weeks after vaccination, were elderly (>75-year-old), and 2 of them had a history of metastatic cancer.

### 3.3. Effect of Prior Vaccination on Hospitalization Outcomes

**Figure 1:**
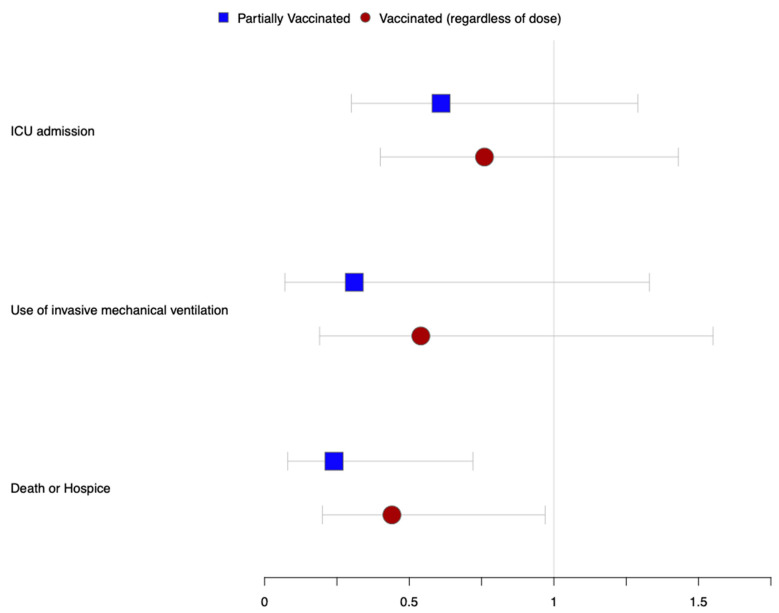
Association of prior COVID vaccination with hospitalization outcomes.

On our multivariate logistic regression analyses, the previous receipt of vaccination, regardless of the dose or days since vaccination, was associated with decreased mortality (aOR 0.44, 95% CI: 0.20–0.98). Even a single dose of mRNA vaccine was also associated with decreased mortality (aOR 0.24, 95% CI: 0.08–0.72) when compared to unvaccinated individuals. Finally, although patients with prior vaccination had lower odds of mechanical ventilation and ICU admission, those associations did not reach statistical significance (Figure 1 and Table 3).

## 4. Discussion

Previous studies have suggested reduction in SARS-CoV-2 infection rates following vaccination [5], especially in fully vaccinated patients (14 days after completing the vaccine series). This 2-week period after vaccination is the CDC accepted standard for individuals to be considered vaccinated and generate an optimal antibody response [6]. However, in our study, even partial vaccination was strongly associated with decreased mortality (aOR 0.24, 95% CI: 0.08–0.72) among hospitalized patients with COVID-19. In the latest report by CDC for the same time period (January–April 2021), a total of 10,262 vaccine breakthrough cases were identified across the US. Among those cases 706 patients (~6.8%) were hospitalized with symptomatic COVID-19, and 132 patients died [4].

Our findings also highlight that populations with potential suboptimal immunogenicity to the vaccine, such as elderly adults [7], continue to be at risk for fatal infection, and strong adherence to infection prevention measures is warranted even in the setting of full immunization. In addition, populations such as cancer patients [8] and transplant recipients [9] are at higher risk for vaccine breakthrough infections. In this regard, Hall et al. utilized a cohort of 120 transplant recipients and showed that a third dose of mRNA vaccine offered a much higher immune response and neutralizing antibody levels compared to placebo [10]. As of 13th August, CDC recommends that moderately to severely immunocompromised people should receive a third vaccine dose [11].

Although our study did not include the genomic sequence of SARS-CoV-2, vaccine breakthrough infections with SARS-CoV-2 variants should also be expected. A study by Hacisuleyman et al., which included a cohort of 417 fully vaccinated patients in New York State (at least 2 weeks after the second dose of an mRNA-based vaccine) authors reported two cases of vaccine breakthrough infections, with variant sequencing revealing SARS-CoV-2 variants in both of them [12]. Vaccination remains key, and a recent “test-negative design” study by Bernal et al. found that previous vaccine effectiveness estimates were modestly lower against the delta variant. More specifically, effectiveness after two doses of the BNT162b2 was 93.7% against the alpha variant and 88.0% against the delta variant, while after two doses of ChAdOx1 nCoV-19 vaccine, the effectiveness was 74.5% against the alpha variant and 67% against the delta variant [13]. However, as the pandemic evolves, SARS-CoV-2 adaptation to vaccine-induced neutralizing antibodies and the development of further escape mutants pose a great public health threat. The role of vaccination in the development of escape mutants merits further study [14].

Limitations of this study include the small sample size of hospitalized vaccine breakthrough cases, its retrospective nature, and the possible lack of generalization, especially given the emergence of mutant strains. In addition, genetic sequence and neutralizing antibodies titers were not available. However, patients included in this study are of high clinical and public health significance as we excluded those who were hospitalized for a reason unrelated to COVID-19 and were tested positive while completely asymptomatic. A similar report strategy is also followed by CDC as of 1 May 2021 [4].

## 5. Conclusions

Despite the presence of vaccine breakthrough COVID-19 cases, the present brief report underlines the importance of vaccination against COVID-19. In our study, even a single dose of vaccination was associated with decreased risk of death. As of August 2021, 72% of the U.S. adult population has received at least one dose of the COVID vaccine [15], while more than 1.7 billion vaccine doses have been administered worldwide [16]. The need for ongoing mass vaccination efforts is still of utmost importance, while vaccination in middle- and low-income countries should remain an absolute priority [16].

## Figures and Tables

**Table 1:** Patient Baseline Characteristics.

	Not Vaccinated	1 Dose of mRNA-Based Vaccine	2 Doses of mRNA-Based Vaccine	1 Dose of J&J	*p*-Value
	*n* = 824	*n* = 75	*n* = 8	*n* = 8	
Patient Age In Years	67.00 (56.00–78.00)	72.00 (63.00–85.00)	78.00 (75.50–81.50)	66.50 (58.00–76.00)	0.002
Patient Sex					0.19
Female	399 (48.4%)	43 (57.3%)	6 (75.0%)	3 (37.5%)	
Male	425 (51.6%)	32 (42.7%)	2 (25.0%)	5 (62.5%)	
Race					0.26
Hispanic or Latino	157 (19.1%)	5 (6.7%)	1 (12.5%)	2 (25.0%)	
Non-Hispanic Black	70 (8.5%)	9 (12.0%)	0 (0.0%)	1 (12.5%)	
Non-Hispanic White	529 (64.2%)	56 (74.7%)	7 (87.5%)	5 (62.5%)	
Other/Unknown	68 (8.3%)	5 (6.7%)	0 (0.0%)	0 (0.0%)	
Congestive heart failure	119 (14.4%)	9 (12.0%)	1 (12.5%)	2 (25.0%)	0.78
Cardiac arrhythmias	178 (21.6%)	18 (24.0%)	2 (25.0%)	2 (25.0%)	0.96
Hypertension	515 (62.5%)	56 (74.7%)	5 (62.5%)	6 (75.0%)	0.18
Chronic pulmonary disease	190 (23.1%)	17 (22.7%)	3 (37.5%)	2 (25.0%)	0.81
Diabetes	283 (34.3%)	28 (37.3%)	3 (37.5%)	5 (62.5%)	0.39
Renal failure	87 (10.6%)	8 (10.7%)	0 (0.0%)	1 (12.5%)	0.81
Liver disease	37 (4.5%)	2 (2.7%)	0 (0.0%)	1 (12.5%)	0.54
Obesity	335 (40.7%)	34 (45.3%)	1 (12.5%)	4 (50.0%)	0.31
Immunization Name					
Ad26.COV2.S	-	0 (0.0%)	0 (0.0%)	8 (100.0%)	
mRNA-1273	-	38 (50.7%)	2 (25.0%)	0 (0.0%)	
BNT162b2	-	37 (49.3%)	6 (75.0%)	0 (0.0%)	
Day of infection after 1st Dose of Vaccination					
Days 1–7	-	16 (21%)	-	1 (12%)	
Days 8–14	-	34 (45%)	-	6 (75%)	
Days 15–21	-	15 (20%)	-	1 (12%)	
Day 22 or later	-	10 (13%)	-	0 (0%)	
Day of infection after 2nd Dose of Vaccination					
Days 1–7	-	-	3 (38%)	-	
Days 8–14	-	-	4 (50%)	-	
Days 15–21	-	-	1 (12%)	-	

**Table 2:** COVID-19 outcomes of patients with or without prior vaccination.

	Unvaccinated	Vaccinated with mRNA Vaccine				Vaccinated with Adenoviral Vector Vaccine	
		1–14 days after 1st dose	≥15 days after 1st dose	1–14 days after 2nd dose	≥15 days after 2nd dose	1–14 days after single dose	≥15 days after single dose
	*n* = 824	*n* = 50	*n* = 25	*n* = 7	*n* = 1	*n* = 7	*n* = 1
ICU	149 (18.1%)	6 (12.0%)	3 (12.0%)	3 (42.9%)	0 (0.0%)	1 (14.3%)	0 (0.0%)
Ventilator	67 (8.1%)	1 (2.0%)	1 (4.0%)	1 (14.3%)	0 (0.0%)	1 (14.3%)	0 (0.0%)
Death	98 (11.9%)	3 (6.0%)	0 (0.0%)	3 (42.9%)	0 (0.0%)	1 (14.3%)	0 (0.0%)
Death or Hospice	113 (13.7%)	3 (6.0%)	1 (4.0%)	3 (42.9%)	0 (0.0%)	1 (14.3%)	0 (0.0%)

**Table 3:** Association of prior COVID vaccination with hospitalization outcomes.

* Odds Ratio (95% Confidence Interval)			
	ICU Admission	Use of Invasive Mechanical Ventilation	Death or Hospice
Vaccinated (regardless of dose)	0.76 (0.40–1.43)	0.54 (0.19–1.55)	0.44 (0.20–0.97)
Partially Vaccinated	0.61 (0.30–1.29)	0.31 (0.07–1.33)	0.24 (0.08–0.72)

* Compared to unvaccinated individuals. Adjusted for age, race, gender, obesity, van Walraven comorbidity score, Dexamethasone, and Remdesivir use.
